# Cases of Arsenical Poisoning

**Published:** 1861-08

**Authors:** N. S. Davis

**Affiliations:** Prof. of Practical Medicine, etc.


CASES OF ARSENICAL POISONING, REPORTED TO THE CHICAGO MEDICAL SOCIETY, JULY 19, 1861. BY N. S. DAVIS, M. D., PROF. OF PRACTICAL MEDICINE, ETC.

On the evening of the 8th inst., I was called hastily to a boarding house only a few rods distant from my residence. I found five members of the family, including Mrs. P., the lady of the house and her daughter, two young men boarders, and the cook, all suffering great distress at the epigastrium, active and frequent vomiting, a sense of burning and constriction in the stomach and œsophagus, great prostration, a metallic taste in the mouth, and irregular griping pains in the abdomen. The young lady and one of the young men had nervous twitchings and slight cramps in the muscles of the extremities. On inquiring I learned that the family had taken supper only about half an hour previous, and that all five of these persons began suddenly to be sick within ten or fifteen minutes after eating. Further inquiry developed the fact that some hot muffins had been prepared for supper, of which the five sick persons had eaten, but no others. This led to an immediate examination of the soda and cream of tartar which had been used in making the muffins. Concerning the first I could discover nothing wrong, but the second was evidently not cream of tartar, but a white powder, slowly soluble, distinctly metallic in taste, and very heavy. From the prompt and thorough vomiting in all the cases, I was suspicious that the poison was tartrate of antimony and potassa, and yet the taste and solubility both, differed from that article. A specimen was immediately sent to Prof. Mahla for chemical analysis, and within an hour he returned a message, saying, that the substance sent him, and which had been used as cream of tartar, was unmixed arsenious acid. Accompanying the message was a glass tube with the arsenic reduced to a metallic state, which is here for the inspection of the Society. Though then uncertain as to the nature of the poison taken, I found within a few minutes that all the sick ones had thoroughly evacuated their stomachs by repeated free vomiting, so much so that I was certain nothing could be gained by further evacuants, either by emetics or the stomach-pump. Neither did I believe there was anything left in the stomach that could be neutralized by chemical agents. On the contrary, the symptoms were already those of incipient gastritis with great prostration, and burning pain in the epigastrium. In one case the pain extended to the bowels, and was accompanied by one copious watery evacuation. Hence, I directed warmth to the extremities, sinapisms to the epigastrium, and the sixth of a grain of sulph. morphia, every half-hour, until vomiting ceased, and the patients were inclined to sleep

Their thirst was appeased by pieces of ice which they were allowed to swallow instead of drink. Under this treatment all had been much relieved, both from the vomiting and distress, before midnight, except the young lady. She continued in great distress in the abdomen, with frequent vomiting, a small and frequent pulse, cold extremities, a pallid countenance, and frequent spells of faintness, until 4 o'clock in the morning, when she fell asleep and rested quietly about two hours. She continued to have faintness or sinking spells whenever moved, and occasional vomiting all the subsequent day. The other patients were entirely comfortable so long as they remained at rest. But any attempt to exercise would cause sickness of the stomach, and great muscular weakness. They were all kept quiet for two or three days, allowed only a very bland and simple diet, and all rapidly recovered. In endeavoring to trace the origin of the arsenic, Mrs. P. gave the following statement:—Some three or four weeks previously, a new cook had been engaged for service in the family. Soon after she came, she found fault with the quality of the cream of tartar, which had been procured of their groceryman, and insisted that some should be obtained from a drug store. Accordingly the driver of the groceryman's wagon, the next time he came to deliver packages and receive orders, was requested to procure for them two or three pounds of cream of tartar at some drug store. He pretended to do so, and at his next visit delivered a package, duly labeled, cream of tartar, with the name of some drug store accompanying it. About this time the cook was discharged, and the package was laid aside, unopened,—where it remained until the morning of the 8th inst. In the meantime, the previous supply having been exhausted, Mrs. P. on that morning, opened the package, emptied it into the usual place for cream of tartar, and threw the paper on which the label was pasted, into the fire. She says she is certain that the package was labeled in large and plain letters, "cream of tartar," and she *thinks* the label contained also the name of a drug store, but she cannot remember what store. In the meantime, the young man who took the order and brought the package, has changed his place of employment, and we have not been able to trace it beyond what is indicated in the above statement. It would appear that somebody, dealing in drugs, had been so exceedingly careless as to put up arsenious acid by the pound, in mistake for one of the most common articles in domestic use. Is it possible that any druggist in Chicago, is so careless as to keep large quantities of arsenic in any place where it could possibly be put up in the manner indicated by the above statement? If so, it is high time they were looked after.

